# Sun protection and exposure behaviors among Hispanic adults in the United States: differences according to acculturation and among Hispanic subgroups

**DOI:** 10.1186/1471-2458-12-985

**Published:** 2012-11-15

**Authors:** Elliot J Coups, Jerod L Stapleton, Shawna V Hudson, Amanda Medina-Forrester, Ana Natale-Pereira, James S Goydos

**Affiliations:** 1 The Cancer Institute of New Jersey, 195 Little Albany Street, New Brunswick, NJ, 08901, USA; 2 Department of Medicine, UMDNJ-Robert Wood Johnson Medical School, 125 Paterson Street, New Brunswick, NJ, 08901, USA; 3 Department of Health Education and Behavioral Science, UMDNJ-School of Public Health, 683 Hoes Lane West, Piscataway, NJ, 08854, USA; 4 Department of Family Medicine and Community Health, UMDNJ-Robert Wood Johnson Medical School, One Worlds Fair Drive, Somerset, NJ, 08873, USA; 5 Department of Medicine, UMDNJ-New Jersey Medical School, 185 South Orange Avenue, Newark, NJ, 07103, USA; 6 Department of Surgery, UMDNJ-Robert Wood Johnson Medical School, 125 Paterson Street, New Brunswick, NJ, 08901, USA

**Keywords:** Acculturation, Hispanic, Latino, Skin cancer, Melanoma, Risk behaviors, Sunburn, Prevention

## Abstract

**Background:**

Skin cancer prevention interventions that target the growing number of U.S. Hispanics are lacking. The current study examined the prevalence and correlates of sun protection and exposure behaviors (i.e., sunscreen use, shade seeking, use of sun protective clothing, and sunburns) among U.S. Hispanics with sun sensitive skin, with a focus on potential differences according to acculturation and Hispanic origin.

**Methods:**

The sample consisted of 1676 Hispanic adults who reported having sun sensitive skin (i.e., they would experience a sunburn if they went out in the sun for one hour without protection after several months of not being in the sun). Participants completed survey questions as part of the nationally representative 2010 National Health Interview Survey. Analyses were conducted in August 2012.

**Results:**

Greater acculturation was linked with both risky (i.e., not wearing sun protective clothing) and protective (i.e., using sunscreen) sun-related practices and with an increased risk of sunburns. Sun protection and exposure behaviors also varied according to individuals’ Hispanic origin, with for example individuals of Mexican heritage having a higher rate of using sun protective clothing and experiencing sunburns than several other subgroups.

**Conclusions:**

Several Hispanic subpopulations (e.g., those who are more acculturated or from certain origins) represent important groups to target in skin cancer prevention interventions. Future research is needed to test culturally relevant, tailored interventions to promote sun protection behaviors among U.S. Hispanics. Such initiatives should focus on public health education and increasing healthcare provider awareness of the importance of skin cancer prevention among Hispanics.

## Background

In the United States, the incidence of skin cancer is higher among non-Hispanic white individuals compared to Hispanics (28.7 versus 4.1 per 100,000 annually) [1], but there are considerable disparities in terms of its morbidity and mortality among racial/ethnic minority groups such as Hispanics [2] (i.e., individuals of Central or South American, Cuban, Mexican, Puerto Rican, or other Spanish culture or origin [3]). Compared to non-Hispanic whites, when diagnosed with melanoma, Hispanics are more likely to be diagnosed at a younger age and with more advanced disease and have lower melanoma-specific survival rates [4-9]. The melanoma incidence among Hispanics increased by 19% from 1992 to 2008 [10]. Skin cancer prevention interventions that target specific population subgroups such as Hispanics are lacking. Hispanics are the fastest growing racial/ethnic group in the United States [11] and represent an important population for heightened skin cancer prevention efforts [12-14].

The primary cause of skin cancer across all racial and ethnic groups is excessive exposure to ultraviolet (UV) light [15]. Recommended preventive behaviors include use of sunscreen, staying in the shade, and wearing sun protective clothing. The vast majority of Hispanics do not meet sun protection recommendations [16]. There is a dearth of research examining correlates of sun protection and exposure behaviors among Hispanics. A notable exception is a study of 496 Hispanic adults [16] which found that individuals with a higher level of acculturation to U.S. cultural norms reported a lower likelihood of wearing sun protective clothing and seeking shade and greater use of sunscreen (although the association with sunscreen was not significant in multivariable analyses).

In the current study, we examined the prevalence and correlates of skin cancer-related behaviors in a large sample of Hispanic respondents (*N* = 1676) drawn from the 2010 National Health Interview Survey (NHIS). We extended prior research findings in several ways. As well as focusing on sun protection behaviors (i.e., sunscreen use, shade seeking, use of sun protective clothing), we examined Hispanics’ reported sunburn experiences and hypothesized that acculturation would be positively associated with sunburns. In addition to acculturation, we considered several other potentially important correlates of Hispanics’ sun protection and exposure behaviors, namely Hispanic origin, skin sensitivity to the sun, and occupational sun exposure. Hispanic subgroups from different countries of origin vary widely in their sociocultural backgrounds and differentially engage in various health behaviors [17-20]. However, the extent to which skin cancer-related behaviors vary according to individuals’ Hispanic origin is not known. Skin sensitivity to the sun is a potential confounder of the association between acculturation and sun-safe behaviors among Hispanics [16]. We hypothesized that individuals with more sun sensitive skin would report greater engagement in sun protection behaviors as well as a higher likelihood of experiencing sunburns. The present study focused on Hispanics who reported having sun sensitive skin, as these individuals are at increased risk for developing skin cancer. Occupational sun exposure is an important risk factor for skin cancer [21] and Hispanics are over-represented in several occupations at high risk for sun exposure (e.g., agriculture) [22]. By identifying correlates of sun protection and exposure behaviors among Hispanics, the current study provides valuable insight on the focus and content of future skin cancer prevention interventions for this understudied and rapidly growing population.

## Methods

### Procedure

The NHIS is an annual, nationally representative, cross-sectional survey of non-institutionalized, civilian adults in the United States. Participants are interviewed in English or Spanish in their own homes. The 2010 NHIS is the most recent year of the survey that included all of the variables required for the current study. The 2010 NHIS used a multistage clustered design, with stratification at the state level and oversampling of Hispanic and black populations. The response rate for the data utilized in the current study was 60.8% [23]. Detailed information regarding the NHIS methodology and survey measures is available elsewhere [23]. The current analysis received approval from the University of Medicine and Dentistry of New Jersey (UMDNJ) Institutional Review Board as an exempt study. Analyses were conducted in August 2012.

### Participants

A total of 5158 individuals self-reported Hispanic (or Latino) ethnicity in the 2010 NHIS (by answering the following question affirmatively: “Do you consider yourself to be Hispanic or Latino?”). We excluded individuals for the following reasons: not having sun sensitive skin (that is, their skin would not burn if they went out in the sun for one hour without protection after several months of not being in the sun) (or data missing for this variable) (*n* = 3097); reported not going out in the sun (*n* = 376); diagnosed with skin cancer (or data missing) (*n* = 8); missing data for all of the sun protection and exposure behaviors (*n* = 1). This resulted in an available sample size of 1676 participants.

### Measures

#### Acculturation

We assessed acculturation with two commonly-used proxy measures [24,25]. One measure considered participants’ self-reported nativity and the number of years that individuals born outside of the fifty states of the U.S. had lived in the country. The second measure focused on the language that individuals reported generally using when speaking

#### Sociodemographic factors

Participants reported their Hispanic origin, region of U.S. residence, gender, age, highest level of education, and health insurance coverage.

#### Skin cancer risk factors

Participants indicated whether any first-degree relative had ever been diagnosed with skin cancer. Skin sensitivity to the sun was assessed with an item that asked participants to report what would happen to their skin if, after several months of not being in the sun very much, they went out in the sun for one hour without protection. Participants completed several questions about their current or previous employment. Drawing from prior research [26], we denoted individuals as being at high risk for occupational sun exposure if they reported working in one of the following industries: agriculture; construction; oil or gas extraction; or the postal service (including couriers and messengers).

#### Sun protection and exposure behaviors

Using a 5-point response scale (from *never* to *always*), participants were asked how often they use sunscreen and how often they stay in the shade when outside on a warm sunny day for more than an hour. Using the same response scale, three items asked about the frequency of wearing a wide-brimmed hat, a long-sleeved shirt, and long pants (or other clothing that reaches the ankles) when outside on a warm sunny day for more than an hour. We averaged responses across these three items to create an index of use of sun protective clothing (α = .64). Participants reported the number of times they had experienced a sunburn in the past year (defined as even a small part of the skin turning red or hurting for 12 hours or more). For analytic purposes, we dichotomized responses to the sunburn variable according to whether individuals reported having a sunburn in the past year.

### Statistical analyses

We conducted a series of univariable linear and logistic regression analyses to examine the association between both acculturation and Hispanic origin and each of the sun protection behaviors (continuous outcomes) and having a sunburn in the past year (dichotomous outcome). These were followed by multivariable linear and logistic regression analyses for the same outcomes, with the acculturation, Hispanic origin, sociodemographic, and skin cancer risk factor variables as independent variables. The multivariable analyses allowed us to determine whether associations identified in the univariable analyses were retained after controlling for the sociodemographic and risk factor variables. Very few individuals (1.8%) reported a family history of skin cancer and thus it was not included as an independent variable in the regression analyses. Individuals with missing data for any one variable were excluded from the respective analyses. For all analyses, a cutoff of *p* < .05 was used to determine statistical significance.

## Results

Frequencies for the study variables are shown in Table 1. Just over half (54.0%) of the participants reported being born outside of the United States and 40.6% indicated that they mostly or only used English when speaking. There was a significant association between the two acculturation variables (χ^2^ = 66.3, *p* < .001), such that 69.8% of those born in the United States reported speaking mostly or only English, compared to 19.2% of those (born outside of the United States) residing 15 or more years in the country, 12.8% of those residing 10–14 years, and 8.0% of those living fewer than 10 years in the country. Almost two-thirds of participants reported being of Mexican Hispanic origin. The vast majority of participants resided in the South or West of the country. Over a third (34.8%) of participants reported having no form of health insurance. Twelve percent of participants were denoted as being at high risk for occupational sun exposure. With regard to participants’ reported sun protection and exposure behaviors, almost half (47.1%) never or rarely used sunscreen, 16.8% never or rarely stayed in the shade, and 60.3% never or rarely used sun protective clothing. More than a third of participants (43.1%) reported having a sunburn in the past year.

**Table 1:** Frequencies of study variables

	**Sample %**
Nativity/length of time in the U.S.	
Born in U.S.	46.0
< 10 years in U.S.	11.3
10–14 years in U.S.	8.8
≥ 15 years in U.S.	33.9
Language used when speaking	
Mostly/only Spanish	35.5
Spanish/English equally	23.9
Mostly/only English	40.6
Hispanic origin	
Mexico	62.7
Central/South America	15.3
Puerto Rico	10.9
Cuba	4.3
Other	6.8
Region of U.S. residence	
Northeast	11.6
Midwest	7.2
South	31.8
West	49.4
Female gender	54.3
Age (years)	
18-29	28.9
30-39	24.5
40-49	21.9
50-64	17.2
≥ 65	7.4
Education level	
≤ Some high school	30.9
High school graduate	26.3
Some college	27.4
College graduate	15.5
Health insurance	
Private	45.6
Public	19.7
None	34.8
Family history of skin cancer	1.8
Skin reaction after 1 hour in the sun	
Mild sunburn	49.7
Moderate sunburn	40.2
Severe sunburn	10.1
Risk for occupational sun exposure	
Low	88.0
High	12.0
Use of sunscreen	
Never	36.7
Rarely	10.4
Sometimes	20.6
Most of the time	14.2
Always	18.1
Stay in the shade	
Never	6.3
Rarely	10.5
Sometimes	29.6
Most of the time	32.3
Always	21.4
Use of sun protective clothing^a^	
Never	24.1
Rarely	36.2
Sometimes	21.7
Most of the time	14.6
Always	3.5
Sunburn in the past year	43.1

*Note*. *N* = 1676 Hispanic adults drawn from the 2010 National Health Interview Survey. All percentages are weighted. Data Source: National Center for Health Statistics, 2010 National Health Interview Survey [23]. ^a^For ease of presentation, participants’ scores were rounded to the nearest whole number (from 1 = *never* to 5 = *always*).

### Univariable regressions examining acculturation and Hispanic origin correlates of sun protection and exposure behaviors

Results of the univariable linear and logistic regression analyses are shown in Table 2. Participants with higher levels of acculturation—in terms of being born in the United States or using English as opposed to Spanish when speaking—reported less use of sun protective clothing and a greater likelihood of having a sunburn in the past year. Additionally, individuals who reported using English when speaking reported greater use of sunscreen than those who reported speaking mostly or only Spanish. There were also differences in the sun protection and exposure behaviors according to participants’ Hispanic origin. Individuals of Mexican origin reported more frequent use of sun protective clothing compared to those from Central/South American, Puerto Rico, and Cuba. Lower rates of having a sunburn were reported by individuals from Central/South America and Cuba compared to those from other countries.

**Table 2:** Univariable regression analyses examining acculturation and country of origin correlates of sun protection and exposure behaviors

	**Sunscreen use**		**Staying in the shade**		**Use of sun protective clothing**		**Past year sunburn rate**	
	**b (95% CI)**	**Mean**	**b (95% CI)**	**Mean**	**b (95% CI)**	**Mean**	**OR (95% CI)**	**%**
Nativity/length of time in the U.S.								
Born in U.S.	Reference	2.77	Reference	3.50	Reference***	2.21	Reference***	51.9
< 10 years in U.S.	−0.18 (−0.49, 0.12)	2.59	0.08 (−0.17, 0.32)	3.58	0.25 (0.08, 0.43)	2.46	0.51 (0.34, 0.79)	35.7
10–14 years in U.S.	−0.22 (−0.50, 0.06)	2.55	−0.10 (−0.37, 0.16)	3.40	0.31 (0.06, 0.56)	2.52	0.46 (0.30, 0.71)	33.2
≥ 15 years in U.S.	−0.21 (−0.40, –0.02)	2.56	0.03 (−0.14, 0.19)	3.53	0.31 (0.17, 0.45)	2.52	0.53 (0.41, 0.69)	36.6
Language used when speaking								
Mostly/only Spanish	Reference***	2.39	Reference	3.61	Reference***	2.64	Reference***	30.1
Spanish/English equally	0.31 (0.10, 0.53)	2.70	−0.18 (−0.34, –0.02)	3.43	−0.26 (−0.42, –0.10)	2.39	1.97 (1.42, 2.74)	46.0
Mostly/only English	0.48 (0.28, 0.68)	2.87	−0.11 (−0.26, 0.04)	3.50	−0.51 (−0.65, –0.37)	2.13	2.65 (2.06, 3.41)	53.3
Hispanic origin								
Mexico	Reference	2.63	Reference	3.59	Reference***	2.48	Reference**	44.4
Central/South America	0.04 (−0.21, 0.30)	2.68	−0.23 (−0.42, –0.04)	3.36	−0.22 (−0.41, –0.03)	2.26	0.62 (0.44, 0.88)	33.1
Puerto Rico	0.11 (−0.16, 0.39)	2.75	−0.19 (−0.40, 0.02)	3.40	−0.47 (−0.65, –0.28)	2.02	1.28 (0.84, 1.95)	50.5
Cuba	−0.03 (−0.48, 0.43)	2.61	−0.16 (−0.48, 0.16)	3.43	−0.41 (−0.76, –0.07)	2.07	0.54 (0.30, 0.97)	30.2
Other	0.21 (−0.13, 0.55)	2.84	−0.08 (−0.34, 0.17)	3.50	−0.07 (−0.33, 0.18)	2.41	1.27 (0.80, 2.01)	50.3

*Note*. b = unstandardized regression coefficient; CI = confidence interval; OR = odds ratio. Sunscreen use, staying in the shade, and use of sun protective clothing were each measured on a 5-point scale (1 = *never*, 2 = *rarely*, 3 = *sometimes*, 4 = *most of the time*, 5 = *all of the time*). *N*s from 1619 to 1668 Hispanic adults drawn from the 2010 National Health Interview Survey. Data Source: National Center for Health Statistics, 2010 National Health Interview Survey [23].

**p < .01, ***p < .001, for the association between the correlate and the sun protection behavior (based on a Satterthwaite adjusted *F* test).

### Multiple regressions examining correlates of sun protection and exposure behaviors

Results of the multiple linear regression analyses examining correlates of sunscreen use, staying in the shade, and use of sun protective clothing are shown in Table 3. Sunscreen use did not differ according to individuals’ Hispanic origin or nativity/length of time in the United States. Individuals who reported speaking mostly or only in English had greater sunscreen use than those who indicated that they speak mostly or only in Spanish. With regard to sociodemographic factors, sunscreen use was higher among women, 18–39 year olds (compared to those aged 65 years and older), more highly educated individuals, and those with private health insurance. Participants with the most sun sensitive skin reported using sunscreen more frequently than other individuals. Occupational sun exposure risk was not associated with sunscreen use.

**Table 3:** Multiple regression analyses examining correlates of sun protection and exposure behaviors

	**Sunscreen use**		**Staying in the shade**		**Use of sun protective clothing**		**Past year sunburn rate**	
	**b (95% CI)**	**Adj. Mean**^**a**^	**b (95% CI)**	**Adj. Mean**^**a**^	**b (95% CI)**	**Adj. Mean**^**a**^	**AOR (95% CI)**	**Adj. %**^**b**^
Nativity/length of time in the U.S.								
Born in U.S.	Reference	2.57	Reference	3.52	Reference	2.34	Reference	42.8
< 10 years in U.S.	0.31 (−0.03, 0.66)	2.88	0.07 (−0.21, 0.36)	3.59	0.06 (−0.14, 0.26)	2.40	1.08 (0.61, 1.92)	44.5
10–14 years in U.S.	0.16 (−0.16, 0.49)	2.73	−0.12 (−0.41, 0.17)	3.40	0.08 (−0.14, 0.31)	2.42	0.88 (0.52, 1.52)	40.2
≥ 15 years in U.S	0.14 (−0.08, 0.36)	2.71	0.01 (−0.21, 0.23)	3.53	0.03 (−0.12, 0.17)	2.37	1.08 (0.76, 1.55)	44.5
Language used when speaking								
Mostly/only Spanish	Reference	2.50	Reference		Reference***	2.52	Reference**	
Spanish/English equally	0.20 (−0.03, 0.42)	2.70	−0.11 (−0.30, 0.07)	3.57	−0.10 (−0.25, 0.06)	2.42	1.51 (1.00, 2.26)	34.9
Mostly/only English	0.28 (0.04, 0.51)	2.78	−0.06 (−0.25, 0.13)	3.46	−0.32 (−0.49, –0.15)	2.20	1.96 (1.29, 2.98)	43.8
Hispanic origin								
Mexico	Reference	2.67	Reference	3.52	Reference*	2.41	Reference*	44.0
Central/South America	−0.02 (−0.25, 0.20)	2.64	−0.10 (−0.31, 0.11)	3.42	−0.15 (−0.36, 0.05)	2.25	0.71 (0.50, 1.00)	36.6
Puerto Rico	−0.06 (−0.36, 0.24)	2.60	0.07 (−0.19, 0.33)	3.59	−0.13 (−0.34, 0.07)	2.27	1.26 (0.78, 2.05)	49.2
Cuba	−0.05 (−0.50, 0.41)	2.62	−0.09 (−0.49, 0.31)	3.44	−0.36 (−0.69, –0.02)	2.05	0.51 (0.32, 0.83)	30.1
Other	0.15 (−0.19, 0.48)	2.81	0.10 (−0.20, 0.40)	3.63	0.16 (−0.08, 0.40)	2.56	1.28 (0.72, 2.28)	49.5
Region of U.S. residence								
Northeast	Reference	2.70	Reference**	3.18	Reference***	2.06	Reference	39.0
Midwest	−0.29 (−0.66, 0.09)	2.42	0.25 (−0.07, 0.58)	3.43	0.00 (−0.29, 0.29)	2.06	1.96 (0.98. 3.91)	53.8
South	−0.13 (−0.44, 0.19)	2.58	0.35 (0.11, 0.60)	3.53	0.26 (0.04, 0.48)	2.34	1.14 (0.67, 1.93)	41.9
West	0.05 (−0.25, 0.36)	2.75	0.43 (0.17, 0.68)	3.61	0.46 (0.24, 0.67)	2.51	1.24 (0.73, 2.11)	43.6
Gender								
Male	Reference***		Reference**	3.40	Reference***	2.47	Reference	42.4
Female	0.90 (0.70, 1.10)	2.18	0.22 (0.07, 0.37)	3.62	−0.20 (−0.31, –0.08)	2.27	1.08 (0.82, 1.43)	44.1
Age (years)								
18–29	Reference**	2.78	Reference	3.43	Reference***	2.16	Reference***	52.0
30–39	0.00 (−0.25, 0.25)	2.79	0.12 (−0.07, 0.32)	3.55	0.19 (0.05, 0.33)	2.35	0.92 (0.59, 1.44)	50.2
40–49	−0.17 (−0.44, 0.10)	2.61	0.05 (−0.18, 0.27)	3.48	0.24 (0.07, 0.41)	2.40	0.66 (0.43, 1.01)	42.4
50–64	−0.25 (−0.52, 0.03)	2.54	0.19 (−0.03, 0.41)	3.62	0.31 (0.12, 0.51)	2.47	0.31 (0.20, 0.50)	27.1
≥ 65	−0.62 (−0.96, –0.27)	2.17	0.29 (−0.02, 0.59)	3.71	0.76 (0.53, 0.99)	2.92	0.28 (0.16, 0.47)	24.9
Education level								
≤ Some high school	Reference***	2.48	Reference	3.58	Reference	2.46	Reference*	39.9
High school graduate	−0.02 (−0.26, 0.21)	2.45	−0.03 (−0.24, 0.17)	3.55	−0.13 (−0.29, 0.04)	2.33	1.27 (0.87, 1.85)	45.1
Some college	0.31 (0.08, 0.54)	2.79	−0.14 (−0.35, 0.08)	3.44	−0.16 (−0.33, 0.01)	2.30	1.48 (1.01, 2.17)	48.6
College graduate	0.67 (0.36, 0.97)	3.15	−0.09 (−0.31, 0.12)	3.49	−0.11 (−0.34, 0.12)	2.35	0.89 (0.56, 1.41)	37.4
Health insurance								
Private	Reference***	2.86	Reference	3.52	Reference	2.38	Reference	47.2
Public	−0.26 (−0.49	2.60	0.01 (−0.19, 0.20)	3.52	0.05 (−0.11, 0.21)	2.43	0.78 (0.53, 1.15)	41.7
None	−0.42 (−0.62	2.44	0.01 (−0.18, 0.19)	3.52	−0.07 (−0.22, 0.07)	2.31	0.68 (0.49, 0.93)	38.7
Skin reaction after 1 hour in the sun								
Mild sunburn	Reference***	2.54	Reference***	3.41	Reference***	2.28	Reference**	38.5
Moderate sunburn	0.16 (−0.01, 0.32)	2.70	0.12 (−0.04, 0.28)	3.53	0.09 (−0.02, 0.21)	2.37	1.55 (1.16, 2.07)	48.1
Severe sunburn	0.60 (0.30, 0.90)	3.14	0.57 (0.33, 0.81)	3.99	0.49 (0.28, 0.71)	2.77	1.47 (0.94, 2.31)	46.9
Risk for occupational sun exposure								
Low	Reference	2.65	Reference*	3.55	Reference***	2.29	Reference	43.0
High	0.10 (−0.18, 0.37)	2.75	−0.27 (−0.50, –0.05)	3.28	0.60 (0.42, 0.78)	2.89	1.15 (0.73, 1.81)	46.0

*Note*. b = unstandardized regression coefficient; CI = confidence interval; Adj. = adjusted; AOR = adjusted odds ratio. Sunscreen use, staying in the shade, and use of sun protective clothing were each measured on a 5-point scale (1 = *never*, 2 = *rarely*, 3 = *sometimes*, 4 = *most of the time*, 5 = *all of the time*). *N*s from 1578 to 1616 Hispanic adults drawn from the 2010 National Health Interview Survey. Data Source: National Center for Health Statistics, 2010 National Health Interview Survey [23].

^a^Adjusted means are least squares means obtained from a multiple linear regression analysis. ^b^Adjusted rates of having a sunburn in the past year (%) are predicted marginals obtained from the multiple logistic regression analysis.

*p < .05, ** *p* < .01, *** *p* < .001, for the association between the correlate and the sun protection behavior (based on a Satterthwaite adjusted *F* test).

Participants’ shade seeking did not vary according to the acculturation variables or Hispanic origin. Shade seeking was higher among those residing in the South and West (compared to those in the Northeast), and women, and among those aged 30–39 years or 65 years and older (compared to those aged 18–29 years). Age, level of education, and health insurance coverage were not associated with shade seeking. Individuals with the most sun sensitive skin reported more frequently staying in the shade than those with less sensitive skin. Individuals at high risk for occupational sun exposure were less likely to report staying in the shade.

Participants’ use of sun protective clothing was higher among individuals who reported speaking mostly or only Spanish. Individuals of Mexican origin reported greater use of sun protective clothing than those originating from Cuba. Use of sun protective clothing differed according to U.S. region of residence, with more frequent use reported by individuals living in the West and South and the least frequent use by those residing in the Northeast and Midwest. Use of sun protective clothing was higher among men than women and was also positively associated with age, but it did not differ according to education level or health insurance coverage. Use of sun protective clothing was higher among individuals with the most sun sensitive skin compared to those with less sun sensitive skin. Individuals at high risk for occupational sun exposure reported greater use of sun protective clothing than those at low risk.

The sunburn rate did not vary according to nativity/length of time in the United States, but it was higher among individuals who reported using English as often as, or more often than, Spanish. Higher rates of sunburn were reported by individuals originating from Mexico and Puerto Rico, with those from Central/South American and Cuba having lower rates. Sunburns were more often reported by younger compared to older individuals. Individuals with the least sun sensitive skin had a lower rate of having a sunburn in the past year. Participants’ reported rate of having a sunburn did not differ according to their region of residence, gender, health insurance coverage, or risk for occupational sun exposure.

## Discussion

Consistent with prior research, the results of the current study indicate that many Hispanic adults fail to engage in one or more sun protection behaviors [16,27]. Participants reported more routinely (i.e., most of the time or always) staying in the shade (53.7%) in comparison with sunscreen use (32.3%) and using sun protective clothing (18.1%). A similar pattern has been observed among non-Hispanic white adults in the United States [28]. More than one third of the participants indicated that they never use sunscreen and 43% reported having one or more sunburns in the past year. Results of the univariable analyses indicated that greater acculturation among U.S. Hispanics is linked with both risky (i.e., not wearing sun protective clothing) and protective sun-related practices (i.e., using sunscreen). While there is some epidemiological research linking such behavioral practices with the risk for melanoma, there is more consistent evidence regarding the role that sunburns play as a risk factor for melanoma [29]. We found that around half of the more acculturated Hispanics reported having a sunburn in the past year, which is considerably higher than the approximately one in three rate among less acculturated individuals. While sunscreen use is commonly endorsed by the public and also depicted in the media [30,31], other sun protection behaviors such as staying out of the sun and using sun protective clothing may be more effective in reducing sunburns and the risk for melanoma [28,29,32]. These non-sunscreen behaviors should be strongly emphasized by healthcare providers and in public health initiatives to prevent skin cancer, particularly when targeting more acculturated, English-speaking Hispanics.

The current study provides novel insight on differences in sun protection and exposure behaviors among Hispanics of varying origin. For example, individuals of Mexican origin reported comparably high rates of using sun protective clothing, yet they were more likely to report having a sunburn than Hispanics with origins from several other regions. Participants from Puerto Rico also reported comparatively high rates of having a sunburn. Future research is needed to explore additional sun exposure factors among Hispanics of varying origins, such as the amount of time spent outside during peak hours for UV exposure, which was not assessed in the 2010 NHIS. The fact that sun protection and exposure behaviors differ among Hispanics of varying origins highlights the importance of designing and implementing culturally relevant, tailored skin cancer prevention interventions for Hispanics that take into account heterogeneity among individuals and across population subgroups.

The associations identified in the univariable analyses between the acculturation factors and the sun protection and exposure behaviors were not all retained in the multivariable analyses. This suggests that acculturation may be linked with these behaviors in part due to associations with other sociodemographic, skin sensitivity, or occupational sun exposure factors. Indeed, a number of these factors were significantly associated with Hispanics’ sun protection and exposure behaviors. Consistent with results in the general U.S. population [33,34], as well as a prior study among Hispanic adults [16], Hispanic men reported being less likely to use sunscreen or stay in the shade, but greater use of sun protective clothing than women. However, the sunburn rate did not differ between men and women. Efforts to reduce the sunburn rate among Hispanics may benefit from focusing on different behaviors across gender, such as promoting shade seeking among men and use of protective clothing among women. There were also considerable differences in sun protection and exposure behaviors across age groups. Although individuals aged 65 years and older reported less use of sunscreen than younger adults, they were more likely to seek shade and had a lower sunburn rate. The greater use of sunscreen reported by more educated Hispanics may in part reflect more knowledge about skin cancer prevention [35,36], although future empirical research is warranted in that regard. Hispanics with the most sun sensitive skin were more likely to engage in sun protection behaviors than other individuals, but still reported a considerably higher rate of having a sunburn. These findings are consistent with results among the general U.S. population [36] and strongly suggest that individuals with the most sun sensitive skin should be targeted for skin cancer prevention efforts, regardless of their ethnicity. Although Hispanics at high risk for occupational sun exposure were less likely to seek shade, it is encouraging that they reported greater use of sun protective clothing and had a similar sunburn rate to that of individuals at lower risk for occupational sun exposure. Further research is needed to document and promote sun protection behaviors among Hispanics engaged in occupations that place them at increased risk for sun exposure.

### Limitations

There are several limitations to the current research. Acculturation was assessed using two proxy measures. Although this reflects a practical approach that can be utilized in clinical and public health settings, future research should consider more direct measures of acculturative changes (e.g., bidimensional assessment of attitudes, beliefs, and behaviors with regard to U.S. and Hispanic cultures) [24]. The number of study participants of certain Hispanic origins (e.g., Dominican Republic, Cuba) was relatively low, which although consistent with the U.S. Hispanic population, did not allow us to include them in a separate category (Dominican Republic) or produced wider confidence intervals for the respective associations with the sun protection and exposure behaviors (Cuba). The measures of family history of skin cancer and skin sensitivity to the sun were self-reported, which raises the potential of reporting and other biases. The measure of occupational sun exposure was based on the industry of employment. Future research should consider the type of work performed and the extent to which Hispanics engage in sun protection behaviors during occupational sun exposure.

## Conclusions

Among U.S. Hispanics with sun sensitive skin, more linguistically acculturated individuals and those of Mexican and Puerto Rican origin are at increased risk for experiencing sunburns. These represent important groups to target for future skin cancer prevention initiatives. None of the Hispanic subpopulations uniformly engaged in sun protection behaviors on a regular basis, and thus there is a need to raise awareness of skin cancer risks and preventive measures among Hispanics in general. This will require intervening at multiple levels of the healthcare system, including educating a wide array of healthcare providers about skin cancer prevention among Hispanics, facilitating identification of Hispanics at increased risk for skin cancer, and delivering appropriate prevention messages in the media and public health forums. Future research is needed to develop and test such interventions, which have the potential to reduce the skin cancer-related morbidity and mortality among the rapidly growing population of Hispanics in the United States.

## Competing interests

The authors declare that they have no competing interests.

## Authors’ contributions

EJC conceived and designed the study, conducted the data analyses, lead the data interpretation, and was the primary author of the manuscript. JLS, SVH, AM-F, AN-P, and JSG participated in the design of the study, assisted with the data interpretation, and helped to draft the manuscript. All authors read and approved the final manuscript.

## Pre-publication history

The pre-publication history for this paper can be accessed here:

http://www.biomedcentral.com/1471-2458/12/985/prepub
